# Asymptomatic SARS-CoV-2 Infection in Nursing Homes, Barcelona, Spain, April 2020

**DOI:** 10.3201/eid2609.202603

**Published:** 2020-09

**Authors:** Blanca Borras-Bermejo, Xavier Martínez-Gómez, María Gutierrez San Miguel, Juliana Esperalba, Andrés Antón, Elisabet Martin, Marta Selvi, María José Abadías, Antonio Román, Tomàs Pumarola, Magda Campins, Benito Almirante

**Affiliations:** Vall d’Hebron Hospital Universitari, Barcelona, Spain (B. Borras-Bermejo, X. Martínez-Gómez, M. Gutierrez-San Miguel, J. Esperalba, A. Antón, M.J. Abadías, A. Román, T. Pumarola, M. Campins, B. Almirante);; Universitat Autònoma de Barcelona, Bellaterra, Spain (X. Martínez-Gómez, A. Antón, A. Román, T. Pumarola, M. Campins, B. Almirante);; Servei Atenció Primària Muntanya, Barcelona (E. Martin);; Centre d’Atenció Primària Sant Andreu, Barcelona (M. Selvi)

**Keywords:** nursing homes, elderly, respiratory infections, COVID-19, SARS-CoV-2, prevention and control, infection control, reverse transcription PCR, Spain, severe acute respiratory syndrome coronavirus 2, 2019 novel coronavirus disease, coronavirus disease, zoonoses, viruses, coronavirus

## Abstract

During the coronavirus disease pandemic in Spain, from April 10–24, 2020, a total of 5,869 persons were screened for severe acute respiratory syndrome coronavirus 2 at nursing homes. Among residents, 768 (23.9%) tested positive; among staff, 403 (15.2%). Of those testing positive, 69.7% of residents and 55.8% of staff were asymptomatic.

As of April 2020, Spain was one of the countries accounting for the most coronavirus disease (COVID-19) deaths (*1*). More than half of those deaths occur in persons >80 years of age (*2*), which highlights the vulnerability of the elderly. Moreover, severe acute respiratory syndrome coronavirus 2 (SARS-CoV-2) can be easily spread within nursing homes, causing outbreaks with high associated mortality rate (*3*,*4*). By the beginning of April, the exponential increase of cases overwhelmed the healthcare system in Spain. In this context, rapid outbreak identification and early intervention in nursing homes was needed.

At Vall d’Hebron Hospital, a tertiary hospital in Catalonia, Spain, we conducted test-based screening as a containment measure to promptly implement effective prevention and control measures in nursing homes. We present the early results of a coordinated intervention with primary care teams in ≈6,000 residents and facility staff in nursing homes in our catchment area.

We evaluated 69 nursing homes that had a total census of 6,714 persons. We excluded previous laboratory-confirmed cases of COVID-19. During April 10–24, an integrated team of hospital and primary care staff obtained samples for SARS-CoV-2 testing from all residents and workers: nasopharyngeal and oropharyngeal swab samples both combined in the same collection tube with viral transport media. We used a commercial CE-IVD–marked, real-time reverse transcription PCR–based assay (Cobas SARS-CoV-2; Roche Diagnostics, https://www.roche.com) on a Cobas 6800 system.

Each nursing home director recorded any symptoms present at least 48 hours before the scheduled day of testing for all residents and staff. According to the World Health Organization case definition of a suspected case of COVID-19, a person was classified as symptomatic if fever or acute respiratory symptoms were present at any moment during the preceding 14 days. In the absence of either, the person was considered to be asymptomatic.

We obtained a total of 5,869 samples, 3,214 from residents and 2,655 from facility staff. Overall, 768 (23.9%) residents and 403 (15.2%) staff members tested positive for SARS-CoV-2 (Table). The presence of fever or respiratory symptoms during the preceding 14 days was recorded in 2,624 residents (81.6%) and 1,772 staff members (66.7%). Among those testing positive and for whom we had information about symptoms, 69.7% of the residents and 55.8% of staff were asymptomatic.

**Table:** SARS-CoV-2 test results for residents and staff of 69 nursing homes, Barcelona, Spain, April 2020*

Patient status during the preceding 14 days	SARS-CoV-2 test results, no. (%)†

Residents			Staff	
Positive, N = 768	Negative, N = 2,446	Positive, N = 403	Negative, N = 2,252
Asymptomatic	486 (69.7)	1,727 (89.6)		144 (55.8)	1,311 (86.6)
Symptomatic‡	211 (30.3)	200 (10.4)		114 (44.2)	203 (13.4)

*Results include all residents and staff who were in the facility the day of screening intervention. Testing was by reverse transcription PCR. SARS-CoV-2, severe acute respiratory syndrome coronavirus 2. †Percentage calculated over those with symptom information available; it was missing for 590 (18.4%) residents and 883 (33.3%) staff members. ‡A person was considered symptomatic if fever or respiratory symptoms were present at time of assessment, or at any moment in the preceding 14 days.

On the basis of laboratory results, we planned specific infection prevention and control measures, adapted to facility characteristics in <72 hours. The most relevant measures applied included isolation of infected residents, establishing cohorted areas and designated staff, excluding infected staff from work, ensuring proper supply of personal protection equipment, and training staff about contact- and droplet-based precautions. We established coordinated follow-up evaluation with primary care teams and facility directors.

COVID-19 heavily affected nursing homes, causing uncountable deaths in Spain (*5*,*6*). Restriction policies for visitors in nursing homes were described as part of the state of emergency declared on March 14 (*7*), but a national guideline to reduce the risk for SARS-CoV-2 transmission in these settings was not available until March 24 (*8*). Moreover, despite knowledge of community transmission starting in late February, widespread testing for SARS-CoV-2 was not available until mid-April.

Our data show an overall high prevalence of SARS-CoV-2 infection in residents and staff, noting a high transmission in these settings. Specific aspects of nursing homes (shared rooms or bathrooms, physically or cognitively impaired residents requiring high-demand care, rotating staff working in different facilities) and a limited adoption of prevention and control measures as reported by our teams are some factors that may explain these results. Among those with known symptom status, we found a high proportion of asymptomatic cases: 69.7% of infected residents and 55.8% of infected staff. 

Our study had several limitations. The ascertainment process could lead to misclassification due to atypical symptoms in the elderly. Furthermore, cross-sectional symptom assessment and testing did not allow us to differentiate between presymptomatic and asymptomatic cases. Nevertheless, these values are consistent with a study performed in a nursing facility in King County, Washington, USA, in which 56% of the residents testing positive were asymptomatic (*9*).

Given that presymptomatic and asymptomatic transmission has been demonstrated (*10*), our data suggest that asymptomatic cases could have had an important role in transmission dynamics. Symptoms-based approaches would have failed to correctly identify cases and therefore continued transmission. Furthermore, testing of facility staff should be included as part of the prevention and control measures, because they may contribute to sustained transmission.

In conclusion, the high prevalence of SARS-CoV-2 cases found in nursing homes highlights that this vulnerable population requires special attention and proactive interventions in coordination with the primary care teams. In the context of established community transmission of SARS-CoV-2, we recommend implementing test-based screening irrespective of symptomatology in nursing homes as the best approach to rapidly implement prevention and control measures.
